# Acute Toxicity of Gambierone and Quantitative Analysis of Gambierones Produced by Cohabitating Benthic Dinoflagellates

**DOI:** 10.3390/toxins13050333

**Published:** 2021-05-05

**Authors:** J. Sam Murray, Sarah C. Finch, Jonathan Puddick, Lesley L. Rhodes, D. Tim Harwood, Roel van Ginkel, Michèle R. Prinsep

**Affiliations:** 1 Cawthron Institute, Private Bag 2, Nelson 7042, New Zealand; Jonathan.puddick@cawthron.org.nz (J.P.); Lesley.rhodes@cawthron.org.nz (L.L.R.); tim.harwood@cawthron.org.nz (D.T.H.); roel.vanginkel@cawthron.org.nz (R.v.G.); 2 New Zealand Food Safety Science and Research Centre, Massey University, Private Bag 11 222, Palmerston North 4442, New Zealand; 3 School of Science, University of Waikato, Private Bag 3105, Hamilton 3240, New Zealand; michele.prinsep@waikato.ac.nz; 4 AgResearch, Ruakura Research Centre, Private Bag 3123, Hamilton 3240, New Zealand; sarah.finch@agresearch.co.nz

**Keywords:** ciguatera poisoning, liquid chromatography–tandem mass spectrometry, nuclear magnetic resonance spectroscopy, LD_50_, *Gambierdiscus*, *Coolia*, *Fukuyoa*

## Abstract

Understanding the toxicity and production rates of the various secondary metabolites produced by *Gambierdiscus* and cohabitating benthic dinoflagellates is essential to unravelling the complexities associated with ciguatera poisoning. In the present study, a sulphated cyclic polyether, gambierone, was purified from *Gambierdiscus cheloniae* CAWD232 and its acute toxicity was determined using intraperitoneal injection into mice. It was shown to be of low toxicity with an LD_50_ of 2.4 mg/kg, 9600 times less toxic than the commonly implicated Pacific ciguatoxin-1B, indicating it is unlikely to play a role in ciguatera poisoning. In addition, the production of gambierone and 44-methylgambierone was assessed from 20 isolates of ten *Gambierdiscus*, two *Coolia* and two *Fukuyoa* species using quantitative liquid chromatography–tandem mass spectrometry. Gambierone was produced by seven *Gambierdiscus* species, ranging from 1 to 87 pg/cell, and one species from each of the genera *Coolia* and *Fukuyoa*, ranging from 2 to 17 pg/cell. The production of 44-methylgambierone ranged from 5 to 270 pg/cell and was ubiquitous to all *Gambierdiscus* species tested, as well as both species of *Coolia* and *Fukuyoa*. The relative production ratio of these two secondary metabolites revealed that only two species produced more gambierone, *G. carpenteri* CAWD237 and *G. cheloniae* CAWD232. This represents the first report of gambierone acute toxicity and production by these cohabitating benthic dinoflagellate species. While these results demonstrate that gambierones are unlikely to pose a risk to human health, further research is required to understand if they bioaccumulate in the marine food web.

## 1. Introduction

Ciguatera fish poisoning (CFP) is the most common non-microbial foodborne illness related to finfish consumption in the world [1,2]. It is generally associated with the bioaccumulation of ciguatoxins (CTXs) in the flesh and viscera of fish from all trophic levels. While published estimates of CFP vectors range between 60 [3,4] and 400 species [1], it is the carnivorous fish species that are the most commonly implicated in CFP cases as they are often targeted by commercial and recreational fishermen. In recent years, however, a variety of marine invertebrates including echinoderms (e.g., urchins and starfish), gastropods (e.g., cone snails) and bivalve molluscs (e.g., giant clams) [5,6,7,8,9] have also been identified as CFP vectors. Based on these new findings, the Food and Agriculture Organisation of the United Nations and World Health Organisation (FAO and WHO) expert panel have reclassified this illness as ciguatera poisoning (CP) [10].

The poisoning syndrome is prevalent in all circumtropical regions of the world [11,12], and is particularly prolific throughout the tropical and subtropical waters of the South Pacific Ocean, affecting both populated and remote indigenous island communities [13,14,15]. These communities are intrinsically linked to the reef system for subsistence and trade, which leaves them vulnerable to both the direct and indirect effects of CP [16].

While the existence of CP has been known for centuries [17,18,19], with the first historical event being reported in 1521 [20], the true impact of this illness is not known. It is estimated that 25,000–50,000 people are affected annually, with epidemiological studies indicating that <20% of actual cases are reported [19]. Intoxications manifest as a wide array of symptoms including gastrointestinal discomfort (e.g., vomiting, diarrhoea, nausea), neurological impairment (e.g., inversion of hot and cold, dysaesthesia and paraethesia) and/or cardiovascular complications (e.g., hypotension and bradycardia) [14,21]. Interestingly, differences in symptoms and intrinsic potencies can be geographically assigned, for example, in the Pacific region, neurological symptoms are commonly associated with intoxication events [14,22].

Current thinking is that the causative compounds of CP are produced by the epiphytic, benthic dinoflagellate genus *Gambierdiscus*. This genus of microalgae is found attached to various substrates and the toxins they produce enter the marine food web by herbivorous reef fish grazing on the macroalgae (e.g., in the Pacific region *Gambierdiscus* favours filamentous red and calcareous green species), coralline turfs, dead corals and volcanic sands [23,24,25]. *Gambierdiscus* is regarded as an opportunistic dinoflagellate that proliferates following damage to the reef system from tropical hurricanes, crown of thorn starfish outbreaks or coral bleaching events [26,27]. In addition, the complexity of CP is heightened as *Gambierdiscus* is regularly found in microalgal assemblages with other toxin-producing benthic dinoflagellates from the genera *Amphidinium*, *Coolia*, *Fukuyoa*, *Ostreopsis* and *Prorocentrum* [28].

To date, 18 species of *Gambierdiscus* have been described: *G. australes*, *G. balechii*, *G. belizeanus*, *G. caribaeus*, *G. carolinianus*, *G. carpenteri*, *G. cheloniae*, *G. excentricus*, *G. holmesii*, *G. honu*, *G. jejuensis*, *G. lapillus*, *G. lewisii*, *G. pacificus*, *G. polynesiensis*, *G. scabrosus*, *G. silvae* and *G. toxicus*; although new species are regularly being discovered [29,30,31,32]. This has heightened as global awareness of CP is increasing and research efforts are being focused on this neglected tropical disease. With 18 species, the genus *Gambierdiscus* is one of the largest genera of marine benthic dinoflagellates, and 16 of the reported species have been isolated from the western South Pacific.

**Figure 1:**
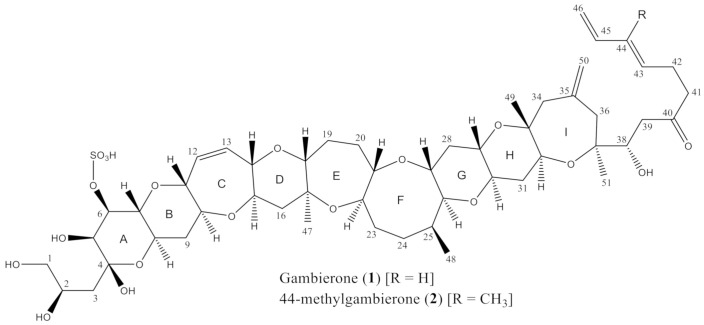
Structures of gambierone and 44-methylgambierone (monoisotopic masses 1024.5 and 1038.5 g/mol, respectively).

The genus *Gambierdiscus* has been shown to produce a complex array of bioactive, ladder-shaped polyether secondary metabolites which have varying levels of toxicity. These include CTXs, which have been demonstrated to biomagnify and biotransform into more toxic analogues as they move up the marine food web, maitotoxins (MTXs), which are some of the most potent non-peptide toxins known, gambieric acids [33], gambierol [34], gambieroxide [35] and gambierones (Figure 1) [36]. While CTXs are thought to be the causative compounds of CP, with the FDA having established a guidance level for finfish of 0.01 µg/kg P-CTX-1B [37], it is currently unclear whether any of the other compounds produced by *Gambierdiscus* play a role in intoxication events.

Determining the toxicity of these secondary metabolites and their abundance in *Gambierdiscus* and cohabitating genera is a critical step in understanding whether they have the potential to cause human illness. Our recent study assessed one of these secondary metabolites, 44-methylgambierone, and determined it had low acute toxicity via intraperitoneal (i.p) injection to mice (LD_50_ between 20 and 38 mg/kg). Its production was also qualitatively assessed in commonly found cohabitating genera of toxin-producing benthic dinoflagellates. The results demonstrated that three genera produced this secondary metabolite: *Gambierdiscus*, *Coolia* and *Fukuyoa* [28].

The work presented in this manuscript expands on this knowledge, where another structurally related secondary metabolite, gambierone, was purified in sufficient quantities to allow for the first determination of its acute toxicity and ascertain if it could play a role in CP events. In addition, quantitative analysis of both gambierone and 44-methylgambierone production by 20 isolates of cohabitating dinoflagellates from the genera *Gambierdisucs*, *Coolia* and *Fukuyoa* was performed, as reference material had been generated. 

## 2. Results

### 2.1. Purification and Identification of Gambierone from Gambierdiscus cheloniae CAWD232

Gambierone was purified from *G. cheloniae* CAWD232 monoclonal cultures using a combination of liquid–liquid partitioning, solid-phase clean-up, flash-chromatography and preparative high-performance liquid chromatography.

**Figure 2:**
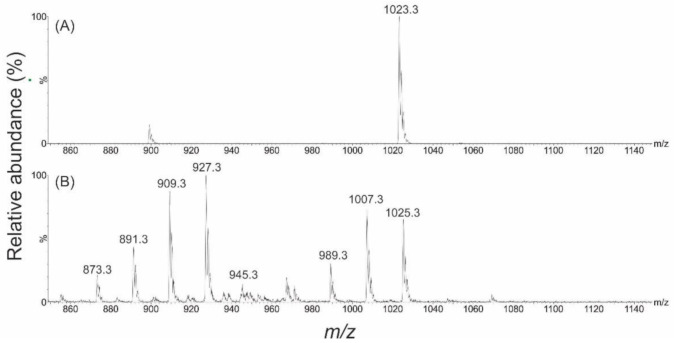
Full scan mass spectra of gambierone (*m/z* 850–1150) in (**A**) −ESI mode showing the [M–H]^−^ ion (*m*/*z* 1023.3) and (**B**) +ESI mode showing the [M+H]^+^ (*m*/*z* 1025.3), [M–H_2_O+H]^+^ (*m*/*z* 1007.3), [M–2H_2_O +H]^+^ (*m*/*z* 989.3) and [M–SO_3_+H]^+^ (*m*/*z* 945.3) ions, as well as a series of sulphite plus sequential water-loss ions (*m*/*z* 927.3, 909.3, 891.3, 873.3).

**Figure 3:**
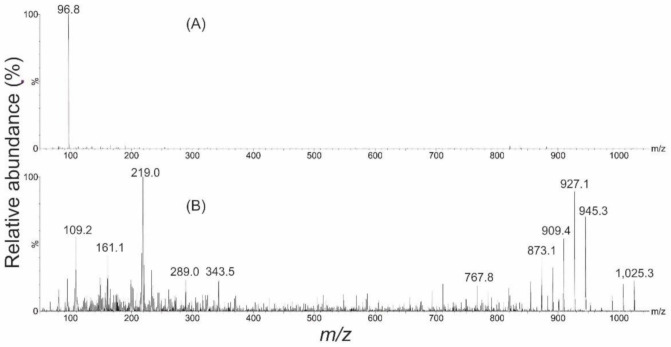
Collision-induced dissociation tandem MS spectra (*m/z* 50–1050) of purified gambierone generated from (**A**) the [M–H]^−^ parent ion (*m*/*z* 1023.3) in –ESI mode, collision energy 70 eV, showing a single dominant fragment ion representing the bisulphate anion (*m*/*z* 96.8) and (**B**) the [M+H]^+^ parent ion (*m*/*z* 1025.3) in +ESI mode, collision energy 25 eV, showing a variety of unassigned fragment ions.

Analysis of the purified gambierone material by electrospray ionization (ESI) mass spectrometry (positive and negative ion modes; *m*/*z* 850–1150), revealed a [M–H]^−^ ion at *m*/*z* 1023.3 and a [M+H]^+^ ion at *m*/*z* 1025.3 (respectively; Figure 2). Additional ions observed in the +ESI spectrum represented water loss (*m*/*z* 1007.3, 989.3) and a [M–SO_3_+H]^+^ ion (*m*/*z* 945.3) plus sequential water-loss ions (*m*/*z* 927.3, 909.3, 891.3, 873.3). These results aligned with those published by Rodriguez et al., 2015 [38] (Supplementary Figure S1). Fragmentation via collision induced dissociation in –ESI mode, revealed a single dominant fragment ion representing a bisulphate anion (*m*/*z* 96.8), and in +ESI there were a variety of unassigned fragment ions (Figure 3).

Analysis of the ^1^H NMR spectrum (Table 1; Supplementary Figures S2–S6) revealed an analogous spectrum, close to that published by Rodriguez et al. [38]. Key signals included those of: the 1,3-diene (H-43 at 5.70 ppm (dt, 15.1, 7.2), H-44 at 6.08 ppm (dd, 15.1, 10.4), H-45 at 6.28 ppm (dt, 16.9, 10.3), H-46a at 5.07 ppm (dd, 17.1, 1.7) and H-46b at 4.94 (dd, 10.3, 1.7)), the connection point of the monosulphate on ring A (H-6 at 4.70 ppm (d, 3.2)) and the terminal diol (H-2 at 4.10 (m)). Other signals observed included those of the alkene protons in ring C (H-12 at 5.64 ppm (dd, 12.5, 2.4) and H-13 at 5.74 ppm (dd, 12.4, 2.5)), the terminal methylene (H-50 at 4.98 (s) and 4.85 ppm (s)) and the methyl groups (H-47 at 1.21 ppm (3H, s), H-48 at 1.00 ppm (3H, s), H-49 at 1.19 ppm (3H, s) and H-51 at 1.13 ppm (3H, s)). For the full comparison of the ^1^H NMR chemical shifts (ppm), multiplicity and coupling constants (Hz) refer to Supplementary Table S1.

In addition to the ^1^H NMR spectrum, COSY (Supplementary Figure S7) and HSQC spectra (Supplementary Figure S8) were acquired. The ^1^H–^1^H and ^1^H–^13^C correlations were also closely aligned to those published by Rodriguez et al. [38] (Supplementary Figures S9–S12). Collectively, the MS and NMR data provided confirmation that the isolated compound was gambierone.

Quantification of the gambierone purified from *G. cheloniae* CAWD232 against a qNMR-calibrated 44-methlygambierone standard using LC–MS/MS, and a relative response factor of 1, determined the yield to be 1.84 mg.

### 2.2. Acute Toxicity of Gambierone by Intraperitoneal Injection

On the basis of the low i.p toxicity previously determined for 44-methylgambierone (between 20 and 38 mg/kg) [28], it was anticipated that gambierone would have a similarly low toxicity. Single mice were dosed at 10, 5, 3.2 and 2.54 mg/kg, all of which died within 4−9 h post-dosing (Table 2). Symptoms of toxicity included abdominal stretching, which was evident 10 min post-dosing. Mice also showed classic signs of discomfort such as pulling their ears back and orbital tightening [39]. As the toxicity progressed the mice became reluctant to move and any movement was jerky. Further deterioration resulted in laboured breathing, and at this point, mice were euthanised to prevent long-term suffering in accordance with the requirements of the Organisation for Economic Cooperation and Development (OECD) Human Endpoints Guidance Document [40]. A single mouse dosed at 2.04 mg/kg gambierone survived, and having established an appropriate dosing range within the OECD guideline 425 [41], the up-and-down method was used to determine the LD_50_ of gambierone as 2.4 mg/kg with 95% confidence intervals of 2.04 and 2.54 mg/kg. All mice dosed at 2.54 mg/kg died in an analogous manner to that described above (Table 2). At necropsy, the small intestine and caecum of each mouse that was administered a lethal dose of gambierone, were found to contain a pale green fluid. All mice dosed at less than 2.04 mg/kg survived. These mice showed abdominal stretching as well as the signs of discomfort mentioned above. All mice appeared normal within 3.5 and 7 h post-dosing. A low food intake and subsequent weight loss over the first 24-h period post-dosing was observed in mice dosed at 2.04 and 1.58 mg/kg. However, for the remainder of the 14-day observation period, food intake and weight gain were normal. This effect on food intake was not noted in mice dosed at rates lower than 1.58 mg/kg. At necropsy, none of the surviving mice showed any abnormalities and the organ weights, as expressed as % of bodyweight, were all within the normal range (data not provided).

### 2.3. Gambierone and 44-Methylgambierone Production by Cohabitating Benthic Dinoflagellates

Dinoflagellate isolates (*n* = 20) were analysed for the production of gambierone and 44-methylgambierone by LC–MS/MS to determine pg/cell quotas. Quantitative analysis was now possible due to the generation of well-characterised reference material for both secondary metabolites. 44-Methylgambierone production was ubiquitous in the ten *Gambierdiscus* species tested, with levels ranging from 26 to 270 pg/cell (Table 3). The highest levels were produced by *G. lapillus* CAWD338 (270 pg/cell) and *G. australes* CAWD149 (259 pg/cell), while the *G. lapillus* CAWD336 isolate produced considerably less (46 pg/cell). Compared to the *Gambierdiscus* species, 44-methylgambierone cell quotas from the *Coolia* and *Fukuyoa* isolates were generally lower (5–65 pg/cell). 

The production of gambierone was more varied, with only seven of the *Gambierdiscus* species and one each of the *Coolia* and *Fukuyoa* species producing detectable levels (LoQ = 0.01 pg/cell). The gambierone cell quotas for the *Gambierdiscus* isolates ranged from 1 to 87 pg/cell (Table 3), with the highest levels observed in *G. carpenteri* CAWD237 (87 pg/cell). When *G. carpenteri* CAWD237 was grown in K media instead of f/2 media, a higher gambierone quota was observed (87 compared to 65 pg/cell). Similar to what was observed for 44-methylgambierone production, the range and level of gambierone cell quotas by the *Coolia* and *Fukuyoa* isolates was lower (2–17 pg/cell). The largest variation was observed between the two *C. malayensis* isolates CAWD154 and CAWD175 (2 and 17 pg/cell, respectively). Gambierone was not detected in the two *C. tropicalis* or the two *F. paulensis* isolates.

The relative production ratio, calculated as pmol/cell, between gambierone and 44-methylgambierone was varied and ranged from 0.01 to 2.15. Of the 14 microalgal species tested, only two produced more gambierone than 44-methylgambierone; *G. carpenteri* CAWD237 (1.19 in K media and 1.46 in f/2 media) and *G. cheloniae* (2.15). Of the isolates where gambierone was detected, the lowest levels of relative production were observed for *G. lewisii* CAWD369 and *G. pacificus* CAWD337, both of which had a ratio of 0.01. 

## 3. Discussion

In the present study, *G. cheloniae* CAWD232 was grown (100 L) to purify enough gambierone to determine its acute toxicity and ascertain if it could play a role in CP events. Fractionation of a methanolic extract and subsequent purification was tracked using LC–MS/MS. The compound was identified as gambierone through its mass spectral properties (the deprotonated and protonated molecular ions; the observed sulphate-/water-loss ions characteristic of cyclic polyethers) and comparison of NMR spectroscopic data with those previously published by Rodriguez et al. in 2015 [38]. The chemical shifts, coupling constants and overlaying of the ^1^H, COSY and HSQC NMR spectra provided unequivocal evidence that the isolated compound was gambierone. As the HSQC spectrum was used to assign the ^13^C chemical shifts, quaternary carbons were unable to be assigned using the current dataset. Small differences in the peak resolution, chemical shifts and coupling constants between the two studies were observed and are a result of the ^1^H NMR data in the current study being acquired at 500 MHz, and the Rodriguez et al., 2015 [38] data being acquired at 750 MHz. 

Due to the structural similarities between gambierone and 44-methylgambierone (i.e., the presence of a monosulphate, terminal diol and 1,3-diene) the quantity of gambierone generated was determined by LC–MS/MS against a qNMR reference standard of 44-methylgambierone [28,36]. A relative response factor of 1 was used as the only structural difference between these two compounds is an additional methyl group on C-44, which is unlikely to grossly affect its ionisation properties. Analysis was performed in –ESI mode for two reasons: (1) it afforded a clean spectrum with good sensitivity and (2) the charge is on the sulphate group, which is at the opposite end of the molecule to the additional methyl group, hence, it would not yield a noticeable effect on the ionisation efficiency of the two compounds. 

Based on the low acute i.p toxicity of 44-methylgambierone (between 20 and 38 mg/kg) [28], it was anticipated that gambierone would also have a low acute toxicity. The LD_50_ of gambierone was found to be 2.4 mg/kg, indicating that it is 10–15 times more toxic than 44-methylgambierone. Given that the only structural difference between these two compounds is the addition of a methyl group on C-44, this difference in toxicity is surprising. It also highlights that toxicity cannot be predicted on the basis of structure, and that small structural changes have the potential to greatly affect toxicity. This may be due to the structural change affecting the three-dimensional shape of the molecule such that its access to the active site responsible for the toxic effect is altered. While the i.p toxicity data of gambierone is new and important information, oral dosing is the most relevant administration route to assess the risk posed to humans. Previous studies on other shellfish toxins have shown that, although toxicity is reduced when given orally rather than by i.p, there is no correlation between the two. For example, among the paralytic shellfish toxins, the difference between oral and i.p toxicity ranges from 30-fold for decarbamoyl neosaxitoxin to 400-fold for gonyautoxin 5 [42]. These differences are likely due to differences in metabolism or absorption of the metabolites, parameters which are bypassed when the compounds are administered by i.p. Unfortunately, it was not possible to isolate the quantities of gambierone that would be required to determine an oral LD_50_. 

Although the i.p LD_50_ of gambierone showed it to be more toxic than 44-methylgambierone, an LD_50_ of 2.4 mg/kg is still indicative of low toxicity. This is consistent with results published by Rodriguez et al. [38], who showed that gambierone had low activity in sodium channels and little impact on cytosolic Ca^2+^ levels in the neuroblastoma SH-SY5Y cell line. In comparison, other toxins implicated in CP are of far greater toxicity. Algal CTXs have i.p LD_50_ values of 2−10 µg/kg and fish metabolite CTXs have i.p LD_50_ values of 0.25−0.45 µg/kg, thereby ranging from being 240 (for 10 µg/kg) to 9600 (for 0.25 µg/kg) times more toxic than gambierone [43]. Although further work is required to assess the bioaccumulation of this secondary metabolite in the marine food web, the hydrophilic nature of gambierone along with the vast difference in toxicity compared to CTXs mean that it is highly unlikely that gambierone contributes to CP. The symptoms of gambierone intoxication are also significantly different to those observed for the CTXs. CTX toxicity is characterised by diarrhoea and hypersalivation, neither of which were observed in this study. A comparison of the symptoms induced by gambierone and 44-methylgambierone is limited, since only one mouse dosed with 44-methylgambierone (38 mg/kg, a lethal dose) showed toxicity. However, it is noted that this mouse did not show the abdominal stretching that was consistently seen in mice dosed with gambierone, although both toxins affected food intake. In addition, this effect was limited to 1−2 days for gambierone, whereas a much more pronounced anorexic effect was seen in the one mouse given a lethal dose of 44-methylgambierone [28]. Although there are some apparent differences in symptomology between gambierone and 44-methylgambierone, additional toxicity work with the latter compound is required for confirmation.

Previous research on 44-methylgambierone production showed that it was ubiquitous to all *Gambierdiscus* species tested [28]. The current work has expanded on this by demonstrating that *G. holmesii* CAWD368 and *G. lewisii* CAWD369 also produce 44-methylgambierone, and by assessing the gambierone production in a range of *Gambierdiscus*, *Coolia* and *Fukuyoa* isolates (*n* = 20). These isolates were sourced from the Cawthron Institute Culture Collection of Microalgae (CICCM) or were donated from research collaborators in Hong Kong and Australia. The *Gambierdiscus* isolates were collected in Australia, the Cook Islands, French Polynesia, the Kermadec Islands and the Federated States of Micronesia. Representative isolates from the ten species held in culture—*G. australes*, *G. caribaeus*, *G. carpenteri*, *G. cheloniae*, *G. holmesii*, *G. honu*, *G. lapillus*, *G. lewisii*, *G. pacificus* and *G. polynesiensis*—were selected. Two isolates were selected for both *G. lapillus* and *G. polynesiensis*, and for *G. carpenteri* the selected isolate was grown in two different growth media to investigate if differences in the production of secondary metabolites would be observed. The four *Coolia* isolates from two species, *C. malayensis* and *C. tropicalis*, and four *Fukuyoa* isolates from two species, *F. paulensis* and *F. ruetzeri,* were selected as they had previously been shown to produce 44-methylgambierone [28].

A quantitative analysis of 44-methylgambierone and gambierone was also undertaken in the current study to evaluate cell quotas. Production of 44-methylgambierone varied between the dinoflagellate isolates, with *G. lapillus* CAWD338 producing the most. Interestingly, the second highest producer was *G. australes* CAWD149, which is the only known MTX-1 producer [36]. Our previous work with *G. carpenteri* isolates from around the Pacific region reported this species to produce 44-methylgambierone, except in the case of *G. carpenteri* CAWD237 collected in Australia [28]. In contrast, the present study demonstrated that the isolate does produce this secondary metabolite. However, because the original analysis was performed on a cell pellet grown at a different laboratory, it is possible that the different seawater/growth medium source, along with different growth cabinets (light intensity, temperature controls), affected the production of this secondary metabolite. This suggests that culturing conditions play a critical role in the production of these compounds, but further investigations are required to properly understand this.

The production of gambierone by *Gambierdiscus* species was varied, with only seven species producing detectable levels (>0.01 pg/cell; *G. carpenteri*, *G. cheloniae*, *G. holmesii*, *G. honu*, *G. lewisii*, *G. pacificus* and *G. polynesiensis*). *G. carpenteri* CAWD237 was the highest producer followed by *G. cheloniae* CAWD232, which was the isolate used for the purification of gambierone in the present study. To date, *G. belizeanus* has been the only species reported to produce gambierone, thereby making this the first report of gambierone production by an additional seven *Gambierdiscus* species.

An interesting observation was made with the *G. carpenteri* CAWD237 isolate, in that it produced more gambierone and 44-methylgambierone when grown in K media compared to f/2 media. This is unusual, as it is well known that the preferred growth medium for *Gambierdiscus* is f/2 media [44]. K media has been specifically designed for growing marine dinoflagellates that cannot survive in higher levels of trace metals. One potential explanation is that this culture had reduced metal availability resulting in a stressed state [45], which in turn increased the production rates of these secondary metabolites. Further research with the remaining isolates is required to test this hypothesis, as well as the effects of the presence/absence of inorganic phosphates on growth and toxin production [45].

Both strains of the two *Coolia* species, *C. malayensis* CAWD154 and CAWD175 and *C. tropicalis* UTS2 and UTS3, produced 44-methylgambierone, whilst only the *C. malayensis* isolates produced gambierone (2 and 17 pg/cell). A similar result was observed with both strains of the two *Fukuyoa* species, *F. paulensis* CAWD238 and CAWD306 and *F. ruetzeri* S044 and S051, producing 44-methylgambierone, while only the *F. ruetzeri* isolates produced gambierone (6 and 8 pg/cell). This is the first report of gambierone production by the genera *Coolia* and *Fukuyoa*. 

Investigation of the production ratio between gambierone and 44-methylgambierone across all species revealed that only two species produced more gambierone than 44-methylgambierone. The first was *G. cheloniae* CAWD232, which produced more than twice the amount of gambierone compared to 44-methylgambierone (ratio of 2.12), followed by *G. carpenteri* CAWD237 (ratio of 1.17 and 1.44 for K and f/2 media, respectively). The largest inverse variation was observed for *G. lewisii* CAWD369 and *G. pacificus* CAWD337 (both with a ratio of 0.01).

While this is the first study to accurately quantify gambierone and 44-methylgambierone production by *Gambierdiscus* species, little research has been conducted to accurately quantify cells quotas using LC–MS/MS for the additional cyclic polyethers produced by this genus. The few that have been reported show that *G. australes* isolates can produce MTX-1 at 2−9 pg/cell, and *G. polynesiensis* CAWD212 produces total CTXs (sum of P-CTX-3B, P-CTX-3C, P-CTX-4A and P-CTX-4B) at 0.44 pg/cell [46]. One research group demonstrated that *G. polynesiensis* TB92 produced 11.9 ± 0.4 pg P-CTX-3C equivalence/cell, as determined using the receptor-binding assay. Subsequent analysis using LC–MS/MS revealed that five CTX analogues were present (P-CTX-3B, P-CTX-3C, P-CTX-4A, P-CTX-4B and M-seco-CTX-3C); however, they were not quantified [13].

## 4. Conclusions

*Gambierdiscus* produces a complex array of bioactive ladder-shaped polyether secondary metabolites. Understanding the toxicity of these compounds is essential in determining if they contribute to CP. One of these compounds, gambierone, was purified from *G. cheloniae* CAWD232 and found to have low acute toxicity by i.p injection in mice (LD_50_ 2.4 mg/kg). The hydrophilic nature of this secondary metabolite and the low acute toxicity compared to CTXs, 9600 times less toxic than P-CTX-1B, indicate that gambierone is unlikely to play a role in CP. However, to confirm this prediction, further research is required to assess the bioaccumulation of this secondary metabolite in the marine food web. In addition, cell quotas were determined and seven of the ten *Gambierdiscus* species tested produced gambierone, along with one species from the genera *Coolia* and *Fukuyoa*. The cell quotas of another structurally related analogue, 44-methylgambierone, were also determined and ubiquitous production by all *Gambierdiscus* species, as well as both the *Coolia* and *Fukuyoa* species tested, was demonstrated. This is the first report of the acute toxicity of gambierone and the quantitative analysis of gambierones produced by these genera of cohabitating benthic dinoflagellates.

## 5. Materials and Methods

### 5.1. Purification of Gambierone

#### 5.1.1. Microalgal Culturing

*Gambierdiscus cheloniae* CAWD232 collected from Rarotonga, The Cook Islands, in 2014, was cultured at 25 °C (±2 °C), 40–70 µmol m^−2^ s^−1^ photon irradiance (12:12 h light:dark cycle) [46]. The isolate was grown in f2/seawater (1:3; UV treated and filtered down to 0.22 µm using a Millipore filtration system; Millipore, Toronto, Canada) [47]. Consecutive 5 L monoclonal cultures (total of 100 L), equating to 1.6 × 10^8^ cells, were harvested during the stationary phase of the growth cycle by centrifugation (3200× *g*, 10 °C, 10 min; Eppendorf 5810 R, Hamburg, Germany).

#### 5.1.2. Extraction and Isolation

The cell pellets from Section 5.1.1. were frozen (−20 °C) before being extracted three times with 90% aq. methanol (MeOH; Fisher-Optima), at a ratio of 1 mL per 2 × 10^5^ cells, and ultrasonication (10 min at 59 kHz; model 160HT, Soniclean Pty, Adelaide, Australia). Cellular debris was pelleted by centrifugation (3200× *g*, 4 °C, 5 min) between extractions and the supernatant combined in a Schott bottle. To remove lipids, the 90% aq. MeOH extract was subjected to a liquid–liquid partition with *n*-hexane (1:1, *v*/*v*; Thermo-Fisher). The 90% aq. MeOH layer was collected and frozen (−20 °C) to precipitate extracellular co-extractives, followed by centrifugation (3200× *g*, 4 °C, 10 min) and sequential membrane and glass-fibre filtration (8, 2 and 1.6 µm) to remove any fine particulates. 

The extract was then diluted to 60% aq. MeOH before completing a second liquid–liquid partition with dichloromethane (DCM; 1:1, *v*/*v*; HiPerSolv Chromanorm, VWR International) to remove any remaining lipophilic compounds. The 60% aq. MeOH containing gambierone was collected, dried down using rotary-evaporation (50 mBar and 50 °C), resuspended in 30 mL Milli-Q water (18.2 MΩ; Millipore, Toronto, ON, Canada) and loaded onto a Strata-X prepacked solid-phase cartridge (10 g; Phenomenex; Torrance, CA, USA). The column was conditioned with ethanol, MeOH and then Milli-Q water (200 mL of each) and washed with 40% aq. MeOH (200 mL). The gambierone was eluted with 100% MeOH (200 mL).

Fractionation was then performed on a Reveleris Flash Chromatography system (Büchi, Flawil, Switzerland) fitted with an Agilent Superflash C_18_ SF 25–75 g column (four injections; Santa Clara, CA, USA). The column was eluted at 20 mL/min with (A) Milli-Q water and (B) acetonitrile (MeCN; Fisher-Optima) mobile phases. The initial solvent composition was 20% B for 5 min before a linear gradient to 95% B from 5–30 min, and then held at 95% B for 10 min. Fractions were collected every 30 s (10 mL). A final fractionation was performed on a Shimadzu preparative HPLC system (Kyoto, Japan) using isocratic elution, 35% aq. MeCN with 0.2% (*v*/*v*) of a 25% ammonium hydroxide (NH_4_OH) solution, on a Phenomenex Gemini C18 column (5 µm, 150 × 21 mm; five injections) with a flow rate of 25 mL/min. The fractions containing gambierone were combined, dried down using rotary-evaporation (50 mBar and 50 °C) and resuspended in 100% MeOH (1 mL; for a schematic of the purification scheme refer Supplementary Figure S13).

#### 5.1.3. Nuclear Magnetic Resonance Spectroscopy Evaluation of Gambierone

1- and 2-D NMR spectroscopy experiments were conducted on a Bruker Avance III 500 MHz instrument with a 5 mm BBOF smart probe (Billerica, MA, USA). The instrument was operated at 500 MHz for ^1^H and 125 MHz for ^13^C, with the chemical shifts being determined at 303 K and referenced to the MeOH signal at 3.31 ppm. The gambierone material was taken to dryness under a stream of N_2_ gas at 40 °C, resuspended in d4-MeOH (600 µL, >99.8% deuterium; Sigma-Aldrich, St. Louis, MO, USA) and transferred to a Wilmad^®^ (Vineland, NJ, USA) 5 mm high-precision NMR tube prior to analysis.

#### 5.1.4. Liquid Chromatography–Mass Spectrometry Evaluation of Gambierone

The purified gambierone (from Section 5.1.2) was assessed using a Waters Xevo TQ-S triple quadrupole mass spectrometer coupled to a Waters Acquity UPLC i-Class with a flow-through needle sample manager (Waters; Milford, MA, USA). The mass spectrometer utilized electrospray ionization (positive and negative ion modes) with a scan range of *m*/*z* 850–1150. Chromatographic separation used a Waters Acquity UPLC BEH phenyl column (1.7 μm, 100 × 2.1 mm) held at 50 °C. The column was eluted at 0.55 mL/min using mobile phases containing 0.2% (*v*/*v*) of a 25% NH_4_OH solution in (A) Milli-Q water and (B) MeCN. Initial solvent conditions were 5% B for 1 min with a linear gradient to 95% B from 1.0 to 7.5 min, held at 95% B for 1 min, followed by a linear gradient back to 5% B from 8.5 to 9 min. The column was re-equilibrated with 5% B for 1 min before the next injection. The autosampler chamber was maintained at 10 °C and the injection volume was 1 μL.

### 5.2. Acute Toxicity of Gambierone by Intraperitoneal Injection

#### 5.2.1. Animals

Female Swiss albino mice (18–22 g) were bred at AgResearch, Ruakura, New Zealand. The mice were housed individually during the experiments and were allowed unrestricted access to food (Rat and Mouse Cubes, Specialty Feeds Ltd., Glen Forrest, Western Australia) and water. All experiments were approved by the Ruakura Animal Ethics Committee established under the Animal Protection (code of ethical conduct) Regulations Act, 1987 (New Zealand), Project Number 14988 (approval date 5 March 2020) and Project Number 15296 (approval date 4 March 2021).

#### 5.2.2. Toxicity Assessment

Acute toxicity was determined using the principles of OECD guideline 425 [41]. This guideline employs an up-and-down procedure, whereby one animal is dosed and if it survives, the next mouse receives an increased dose, whereas if it dies, the next mouse is dosed with a reduced amount of the test material. To determine the LD_50_, dosing is continued until four live-death reversals have been achieved.

Toxicity was determined by i.p injection. Each mouse was weighed prior to dosing and the required amount of gambierone calculated to yield the chosen dose on a mg/kg basis. The dose was prepared by taking the appropriate volume of stock solution (pure gambierone in 90% aq. MeOH), drying it down under nitrogen and immediately redissolving in 1% Tween 60 in normal saline (1 mL). This freshly prepared solution was then immediately injected into mice. All dosing was conducted between 8 and 9.30 a.m. to avoid any diurnal variations in response. Mice were monitored closely during the day of dosing and those that survived were monitored for a 14 day observation period, which included a daily measurement of food consumption and bodyweight. After 14 days, the mice were euthanised by carbon dioxide inhalation and necropsied. The weights of the liver, kidneys, spleen, heart, lungs, stomach (full and empty) and the whole gut were measured and calculated as a percentage of bodyweight (data not provided).

### 5.3. Quantitive Analysis of Gambierone and 44-Methylgambierone Production

#### 5.3.1. Microalgal Culturing and Sample Extraction

The microalgal isolates studied (20 in total) consisted of 14 species from three genera, *Gambierdiscus*, *Coolia* and *Fukuyoa*. All isolates were grown in f2/seawater (1:3) except for the *G. carpenteri* isolate, which was also grown in K media. The growth chamber was set at 25 °C (±2 °C), 40–70 µmol m^−2^ s^−1^ photon irradiance (12:12 h light:dark cycle). Isolates were either sourced from the CICCM; or donated by researchers from Hong Kong and Australia. Cultures were harvested in the late exponential or stationary phase and contained at least 5 × 10^5^ cells. The cells were harvested by centrifugation (3200× *g*, 10 °C, 10 min), the growth medium was decanted and the resulting cell pellets were frozen (−20 °C).

Each cell pellet was extracted twice with 90% aq. MeOH, at a ratio of 1 mL per 2 × 10^5^ cells, and ultrasonication (10 min at 59 kHz). Cellular debris was pelleted by centrifugation (3200× *g*, 4 °C, 5 min) between extractions and the supernatant transferred to another vial. The resulting supernatants were pooled to give a final extract concentration equivalent to 1 × 10^5^ cells/mL. The combined extracts were stored at −20 °C for 24–48 h to precipitate insoluble matrix co-extractives, which were removed using centrifugation (3200× *g*, 4 °C, 5 min) prior to analysis. An aliquot of the clarified extract was transferred into a 2 mL glass autosampler vial and analysed using a modification of the LC–MS/MS method described in Murray et al., 2018 [48].

#### 5.3.2. Liquid Chromatography–Tandem Mass Spectrometry Analysis

Quantitative analysis of gambierone and 44-methylgambierone (monoisotopic masses 1024.5 and 1038.5 g/mol, respectively) was performed on a Waters Xevo TQ-S triple quadrupole mass spectrometer coupled to a Waters Acquity UPLC i-Class with a flow-through needle sample manager. Chromatographic separation used a Waters Acquity UPLC BEH phenyl column (1.7 μm, 100 × 2.1 mm) held at 50 °C. The column was eluted at 0.55 mL/min with (A) Milli-Q water and (B) MeCN mobile phases, each containing 0.2% (*v*/*v*) of a 25% NH_4_OH solution. Fresh mobile phases were prepared daily to ensure optimal sensitivity and stable retention times. The initial solvent composition was 5% B with a linear gradient to 50% B from 0 to 2.5 min, ramped up to 95% B by 3 min and held at 95% B until 3.2 min, followed by a linear gradient back to 5% B at 3.5 min. The column was then re-equilibrated with 5% B until 4 min. The autosampler chamber was maintained at 10 °C and the injection volume was 1 μL. The mass spectrometer used an electrospray ionization source operated in negative ion mode. Other settings were capillary voltage 3.0 kV, cone voltage 40 V, source temperature 150 °C, nitrogen gas desolvation flow rate 1000 L/h at 600 °C, cone gas 150 L/h and the collision cell was operated with 0.15 mL/min argon. Multiple reaction monitoring (MRM) transitions for gambierone were *m*/*z* 1023.3 > 96.8 (Channel 1) and *m*/*z* 899.6 > 96.8 (Channel 2), with collision energies of 50 eV. 44-Methylgambierone was monitored using the *m*/*z* 1037.6 > 96.8 (Channel 1) and 899.6 > 96.8 (Channel 2), with collision energies of 70 and 48 eV, respectively. All transitions had a dwell time of 30 ms. Channels 1 and 2 were used for quantitation and confirmation, respectively.

Data acquisition and processing were performed with TargetLynx software (Waters, Milford, MA, USA). Gambierone and 44-methylgambierone were identified in sample extracts based on the retention time (2.54 and 2.58 min, respectively) and a fragment ion ratio of 8:1 (Channel 1/Channel 2; as determined using reference material). Quantitative analysis was performed using a five-point linear regression calibration (0–1000 ng/mL) prepared in 90% aq. MeOH and a relative response factor of 1 to 44-methylgambierone. The LoQ of the analytical method was 1 ng/mL, which equates to 0.01 pg/cell in an extract generated from a cell pellet of 1 × 10^5^ cells/mL.

#### 5.3.3. Quantitation of Gambierone Using Liquid Chromatography–Tandem Mass Spectrometry

The purified gambierone material (Section 5.1.2) was quantified against a qNMR-calibrated 44-methylgambierone reference standard [28] using LC–MS/MS and a relative response factor of 1. The instrument parameters and chromatographic conditions outlined above (Section 5.3.2) were used. Triplicate injections of a 100 ng/mL standard were compared, followed by calibration of the gambierone material using a five-point linear regression calibration (0–1000 ng/mL) of 44-methylgambierone.

## Figures and Tables

**Table 1:** Comparison of the ^1^H (500 MHz) NMR chemical shifts (ppm), multiplicity and coupling constants (Hz) of the key structural signals of gambierone purified from *Gambierdiscus cheloniae* CAWD232 and those published by Rodriguez et al., 2015 [38].

Structural Feature	Atom	Purified Gambierone	Published Gambierone [38]
Terminal diol	2	4.10 (m)	4.11 (m)
Monosulphate	6	4.70 (dd, 10.0, 3.2)	4.70 (dd, 10.0, 3.2)
Alkene in ring C	12	5.64 (dd, 12.5, 2.4)	5.64 (dd, 12.5, 2.5)
	13	5.74 (dd, 12.4, 2.5)	5.75 (dd, 12.5, 2.5)
1,3-diene	43	5.70 (dt, 15.1, 7.2)	5.70 (dt, 15.0, 7.0)
	44	6.08 (dd, 15.1, 10.4)	6.08 (dd, 15.0, 10.5)
	45	6.28 (dt 16.9, 10.3)	6.29 (dt, 17.0, 10.4)
	46a	5.07 (dd, 17.1, 1.7)	5.08 (dd, 17.0, 1.8)
	46b	4.94 (dd, 10.3, 1.7)	4.94 (dd, 10.3, 1.8)
Methyl group	47	1.21 (3H, s)	1.20 (3H, s)
	48	1.00 (3H, d, 7.3)	1.00 (3H, d, 7.3)
	49	1.19 (3H, s)	1.19 (3H, s)
Terminal methylene	50	4.98 (br s), 4.85 (br s)	4.98 (br s), 4.86 (br s)
Methyl group	51	1.13 (3H, s)	1.13 (3H, s)

**Table 2:** Lethality, death time, recovery time and symptoms of toxicity following acute i.p. injection of gambierone to mice.

Dose (mg/kg)	Lethality	Death (h)	Recovery (h)	Symptoms of Toxicity
1.00	0/1		4 ½	Abdominal stretching
1.26	0/1		7	Abdominal stretching
1.58	0/1		3 ½	Abdominal stretching, ears back, low food intake for 1 day
2.04	0/3		3–3 ½–4 ½	Abdominal stretching, ears back, orbital tightening, low food intake for 1 day
2.54	2/2	5 ^a^–6 ^a^		Abdominal stretching, ears back, orbital tightening, prostration, jerky movement, laboured breathing
5.00	1/1	9 ^a^		Abdominal stretching, ears back, orbital tightening, prostration, jerky movement, laboured breathing
10.0	1/1	4		Abdominal stretching, ears back, orbital tightening, prostration, jerky movement, laboured breathing

^a^ Mice were euthanised when breathing became laboured to prevent long-term suffering.

**Table 3:** Production of gambierone and 44-methylgambierone in isolates of *Gambierdiscus*, *Coolia* and *Fukuyoa.*

Scientific Name	Culture ID *^a^*	44-Methylgambierone (pg/Cell)	Gambierone (pg/cell)	Production Ratio *^b^*
*G. australes*	CAWD149	259	<0.01	–
*G. caribaeus*	CAWD301	44	<0.01	–
*G. carpenteri* (K media)	CAWD237	74	87	1.19
*G. carpenteri*	CAWD237	45	65	1.46
*G. cheloniae*	CAWD232	26	55	2.15
*G. holmesii*	CAWD368	97	20	0.20
*G. honu*	CAWD242	182	38	0.21
*G. lapillus*	CAWD336	46	<0.01	–
*G. lapillus*	CAWD338	270	<0.01	–
*G. lewisii*	CAWD369	68	1	0.01
*G. pacificus*	CAWD337	100	1	0.01
*G. polynesiensis*	CAWD212	29	13	0.45
*G. polynesiensis*	CAWD267	44	13	0.30
*C. malayensis*	CAWD154	9	2	0.29
*C. malayensis*	CAWD175	24	17	0.72
*C. tropicalis*	UTS2	14	<0.01	–
*C. tropicalis*	UTS3	15	<0.01	–
*F. paulensis*	CAWD238	5	<0.01	–
*F. paulensis*	CAWD306	65	<0.01	–
*F. ruetzeri*	S044	12	8	0.62
*F. ruetzeri*	S051	13	6	0.47

*^a^* Unique laboratory identifier, *^b^* Production ratio of gambierone/44-methylgambierone calculated as pmol/cell (monoisotopic masses 1024.5 and 1038.5, respectively).

## Supplementary Materials

The following are available online at https://www.mdpi.com/article/10.3390/toxins13050333/s1. Table S1. Comparison of the ^13^C (125 MHz) and ^1^H (500 MHz) NMR chemical shifts (ppm), multiplicity and coupling constants (Hz) for gambierone purified from *Gambierdiscus cheloniae* CAWD232, generated in d4-MeOH, and those published by Rodriguez et al., 2015 [38]. Figure S1. Comparison of the mass spectra of gambierone in positive electrospray ionization mode (**A**) purified from *Gambierdiscus cheloniae* CAWD232 and (**B**) published by Rodriguez et al., 2015 [38]. Figure S2. ^1^H NMR spectrum of gambierone purified from *Gambierdiscus cheloniae* CAWD232 acquired on a Bruker Advance III 500 MHz instrument in CD_3_OH (≥99.8% atom D). Figure S3. Expansion of the ^1^H NMR spectrum (0.6–2.7 ppm) of gambierone in CD_3_OD (≥99.8% atom D) at 500 MHz. Figure S4. Expansion of the ^1^H NMR spectrum (2.8–5.2 ppm) of gambierone in CD_3_OD (≥99.8% atom D) at 500 MHz. Figure S5. Expansion of the ^1^H NMR spectrum (5.3–7.3 ppm) of gambierone in CD_3_OD (≥99.8% atom D) at 500 MHz. Figure S6. Comparison of the ^1^H NMR spectrum of (**A**) gambierone purified from *Gambierdiscus cheloniae* CAWD232 acquired on a Bruker Advance III 500 MHz instrument and (**B**) the published spectrum from Rodriguez et al., acquired on a Varian Inova 750 MHz instrument [38]. Figure S7. COSY NMR spectrum of gambierone purified from *Gambierdiscus cheloniae* CAWD232 in CD_3_OD (≥99.8% atom D) at 500 MHz. Figure S8. HSQC NMR spectrum of gambierone purified from *Gambierdiscus cheloniae* CAWD232 in CD_3_OD (≥99.8% atom D) at 500 MHz. Figure S9. Comparison of the COSY NMR spectrum (1.0–6.5 ppm) of gambierone purified from *Gambierdiscus cheloniae* CAWD232 (black) and that published by Rodriguez et al., 2015 (red) [38]. Long range (~2 Hz) couplings not displayed. Figure S10. Expansion (^1^H: 1.0–2.7 ppm; ^13^C: 10–55 ppm) of the HSQC spectrum of gambierone purified from *Gambierdiscus cheloniae* CAWD232 (black), and the published spectrum from Rodriguez et al., 2015 (red) [38]. Figure S11. Expansion (^1^H: 2.5–4.7 ppm; ^13^C: 62–85 ppm) of the HSQC spectrum of gambierone purified from *Gambierdiscus cheloniae* CAWD232 (black), and the published spectrum from Rodriguez *et al*., 2015 (red) [38]. Figure S12. Expansion (^1^H: 4.7–6.5 ppm; ^13^C: 110–140 ppm) of the HSQC spectrum of gambierone purified from *Gambierdiscus cheloniae* CAWD232 (black), and the published spectrum from Rodriguez et al., 2015 (red) [38]. Figure S13. Purification scheme for the isolation of gambierone from *Gambierdiscus cheloniae* CAWD232.
