# Supplementary material for: Acute Toxicity of Gambierone and Quantitative Analysis of Gambierones Produced by Cohabitating Benthic Dinoflagellates
Source: Toxins (Basel). 2021 May 5;13(5):333. doi: 10.3390/toxins13050333 (PMC8147941; doi:10.3390/toxins13050333)
Supplement: Supplementary file 1 [file toxins-13-00333-s001.zip › toxins-1198009-supplementary.pdf]

Supplementary Materials: Acute Toxicity of Gambierone and Quantitative Analysis of Gambierones Produced by Cohabitating Benthic Dinoflagellates

J. Sam Murray, Sarah C. Finch, Jonathan Puddick, Lesley L. Rhodes, D. Tim Harwood, Roel van Ginkel and Michèle R. Prinsep

Table S1. Comparison of the ^{13}C (125 MHz) and ^{1}H (500 MHz) NMR chemical shifts (ppm), multiplicity and coupling constants (Hz) for gambierone purified from *Gambierdiscus cheloniae* CAWD232, generated in d4-MeOH, and those published by Rodriguez *et al*., 2015 [38].

Atom	Purified gambierone		Published gambierone[b]	
	δC[a]	δH (multi, *J* in Hz)	δC	δH (multi, *J* in Hz)
1	67.6	3.47 (-)[c], 3.42 (m)	67.9	3.47 (dd, 11.0, 4.5), 3.42 (m)
2	69.8	4.10 (m)	70.3	4.11 (m)
3	39.6	2.01 (m), 1.70 (dd, 14.5, 10.1)	39.6	2.01 (m), 1.70 (dd, 14.5, 10.0)
4	-[d]	-	101.0	-
5	73.0	4.21 (d, 3.2)	73.1	4.21 (d, 3.2)
6	77.7	4.70 (dd, 10.0, 3.2)	77.9	4.70 (dd, 10.0, 3.2)
7	77.1	3.37 (-)[c]	77.4	3.37 (t, 10.0)
8	67.9	3.76 (m)	68.0	3.77 (m)
9	37.8	2.19 (m), 1.58 (-)[c]	37.8	2.17 (m), 1.58 (q, 12.0)
10	79.6	3.35 (-)[c]	79.8	3.35 (m)
11	82.6	3.80 (m)	82.6	3.79 (m)
12	132.7	5.64 (dd, 12.5, 2.4)	133.2	5.64 (dd, 12.5, 2.5)
13	132.9	5.74 (dd, 12.4, 2.5)	133.5	5.75 (dd, 12.5, 2.5)
14	82.9	3.81 (m)	83.2	3.81 (m)
15	80.0	3.45 (m)	80.0	3.44 (m)
16	47.2	1.98 (m), 1.49 (t, 10.9)	47.4	1.99 (m), 1.49 (t, 11.0)
17	-[d]	-	76.7	-
18	86.8	3.0 (dd, 10.9, 2.5)	87.0	3.00 (dd, 11.0, 2.5)
19	25.1	1.77 (-)[c], 1.62 (-)[c]	25.3	1.78 (m), 1.62 (m)
20	34.0	1.95 (-)[c], 1.81 (-)[c]	34.2	1.95 (m), 1.80 (m)
21	87.3	3.53 (m)	87.6	3.54 (m)
22	75.8	3.53 (m)	75.9	3.54 (m)
23	32.6	1.82 (-)[c], 1.64 (m)	32.7	1.82 (m), 1.64 (m)
24	29.5	1.94 (-)[c], 1.77 (-)[c]	29.5	1.92 (m), 1.77 (m)
25	35.5	2.19 (-)[c]	35.6	2.19 (m)
26	86.0	3.11 (-)[c]	86.1	3.11 (m)
27	77.5	3.51 (m)	77.9	3.51 (m)
28	39.7	2.20 (-)[c], 1.33 (q, 11.2)	39.8	2.20 (m), 1.33 (q,11.3)
29	70.0	3.12 (-)[c]	70.2	3.12 (td, 9.5, 4.3)
30	77.8	2.94 (ddd, 11.8, 9.6, 4.6)	78.3	2.94 (ddd, 11.5, 9.5, 4.5),
31	34.7	1.89 (-)[c], 1.54 (q, 11.7)	34.9	1.90 (m), 1.55 (q, 11.5)
32	72.4	3.78 (m)	72.6	3.77 (m)
33	-[d]	-	77.4	-
34	54.0	2.36 (d, 12.1), 2.14 (-)[c]	54.2	2.35 (d, 12.0), 2.14 (d, 12.0)
35	-[d]	-	144.0	-
36	43.3	2.55 (d, 14.0), 2.22 (d, 14.1)	43.3	2.54 (d, 14.0), 2.22 (d, 14.0)

37	- [d]	-	80.0	-
38	73.1	4.06 (dd, 8.7, 3.6)	73.5	4.06 (dd, 8.7, 3.5)
39	45.7	2.63 (dd, 12.0, 3.0), 2.60 (dd, 12.1, 9.0)	45.8	2.62 (dd, 12.0, 3.0), 2.60 (dd, 12.0, 9.0)
40	- [d]	-	212.2	-
41	43.7	2.61 (t, 7.0)	43.7	2.61 (t, 7.0)
42	27.2	2.33 (q, 7.0)	27.3	2.33 (q, 7.0)
43	134.3	5.70 (dt, 15.1, 7.2)	134.9	5.70 (dt, 15.0, 7.0)
44	132.9	6.08 (dd, 15.1, 10.4)	133.4	6.08 (dd, 15.0, 10.5)
45	138.6	6.28 (dt 16.9, 10.3)	138.9	6.29 (dt, 17.0, 10.4)
46	115.4	5.07 (dd, 17.1, 1.7), 4.94 (dd, 10.3, 1.7)	115.9	5.08 (dd, 17.0, 1.8), 4.94 (dd, 10.3, 1.8)
47	16.2	1.21 (3H, s)	16.2	1.20 (3H, s)
48	13.3	1.00 (3H, d, 7.3)	13.3	1.00 (3H, d, 7.3)
49	16.6	1.19 (3H, s)	16.7	1.19 (3H, s)
50	118.5	4.98 (br s), 4.85 (br s)	119.0	4.98 (br s), 4.86 (br s)
51	20.6	1.13 (3H, s)	20.6	1.13 (3H, s)

[a] Carbon chemical shifts determined from an HSQC spectrum. [b] Values from Rodriguez *et al.*, 2015 [38]. [c] Coupling pattern unable to be determined using current dataset. [d] Quaternary carbon, therefore unable to determine chemical shift from HSQC.

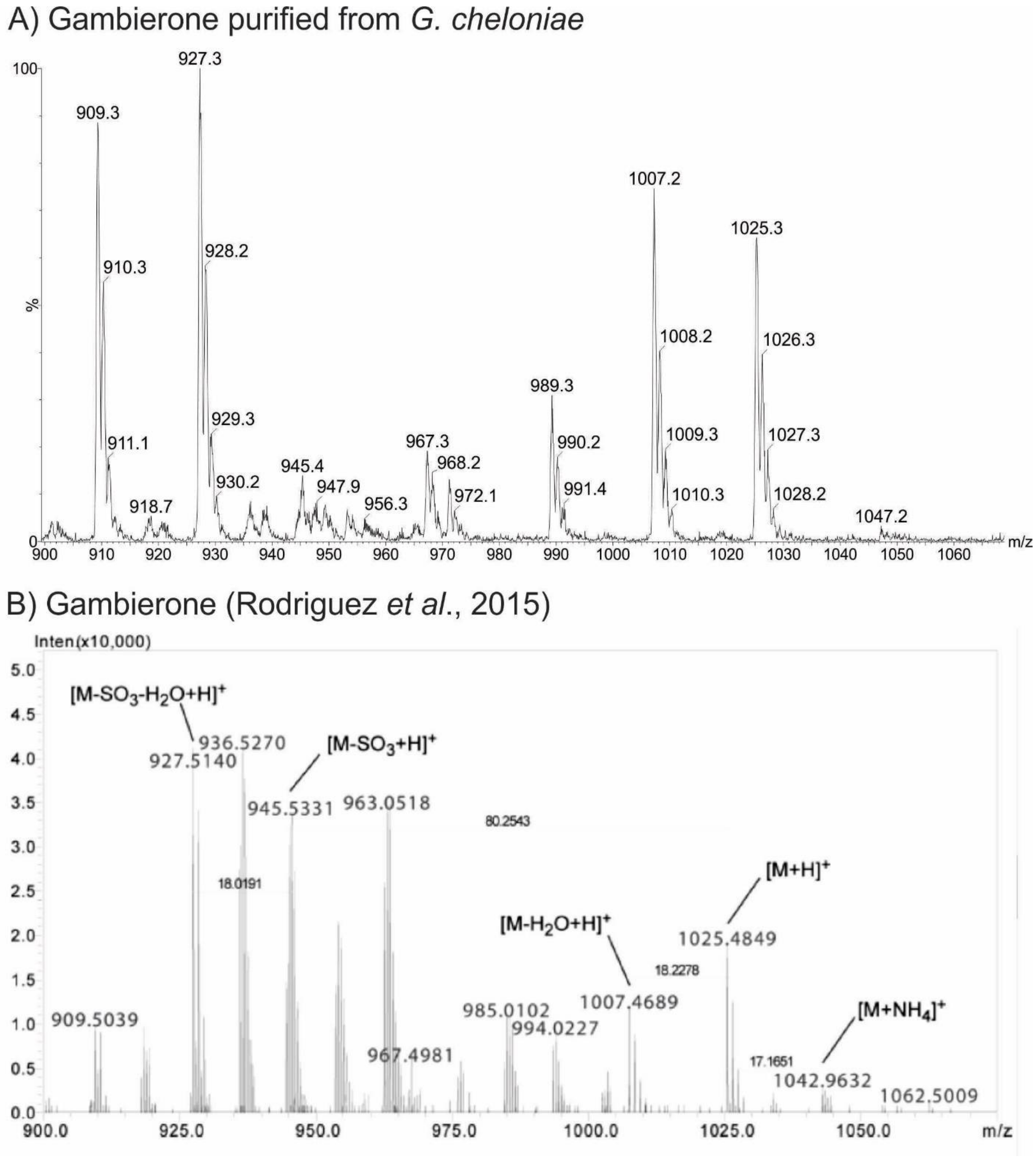

Figure S1. Comparison of the mass spectra of gambierone in positive electrospray ionization mode (**A**) purified from *Gambierdiscus cheloniae* CAWD232 and (**B**) published by Rodriguez *et al.*, 2015 [38].

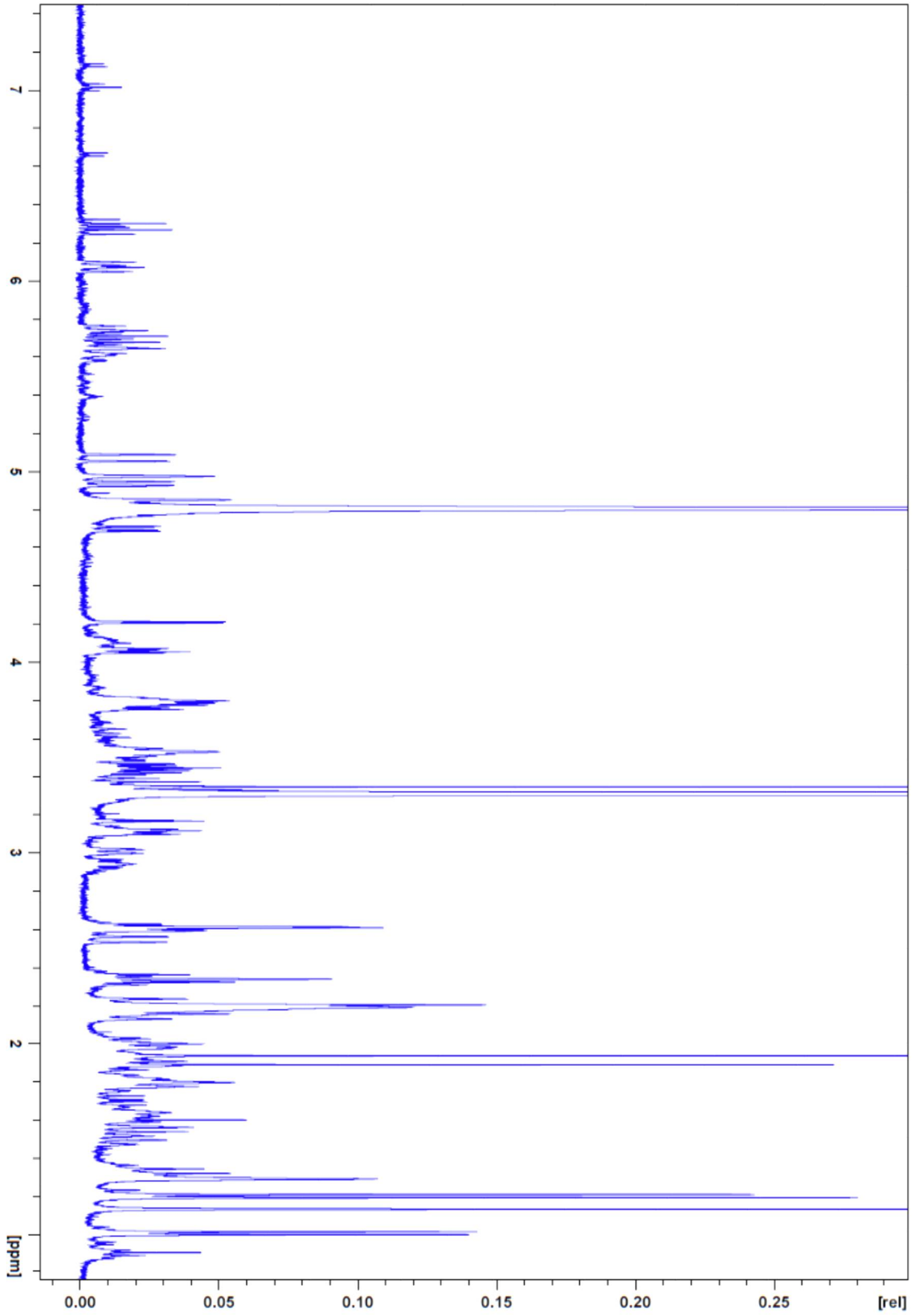

Figure S2. ^{1}H NMR spectrum of gambierone purified from *Gambierdiscus cheloniae* CAWD232 acquired on a Bruker Advance III 500 MHz instrument in CD_3OH (≥99.8% atom D).

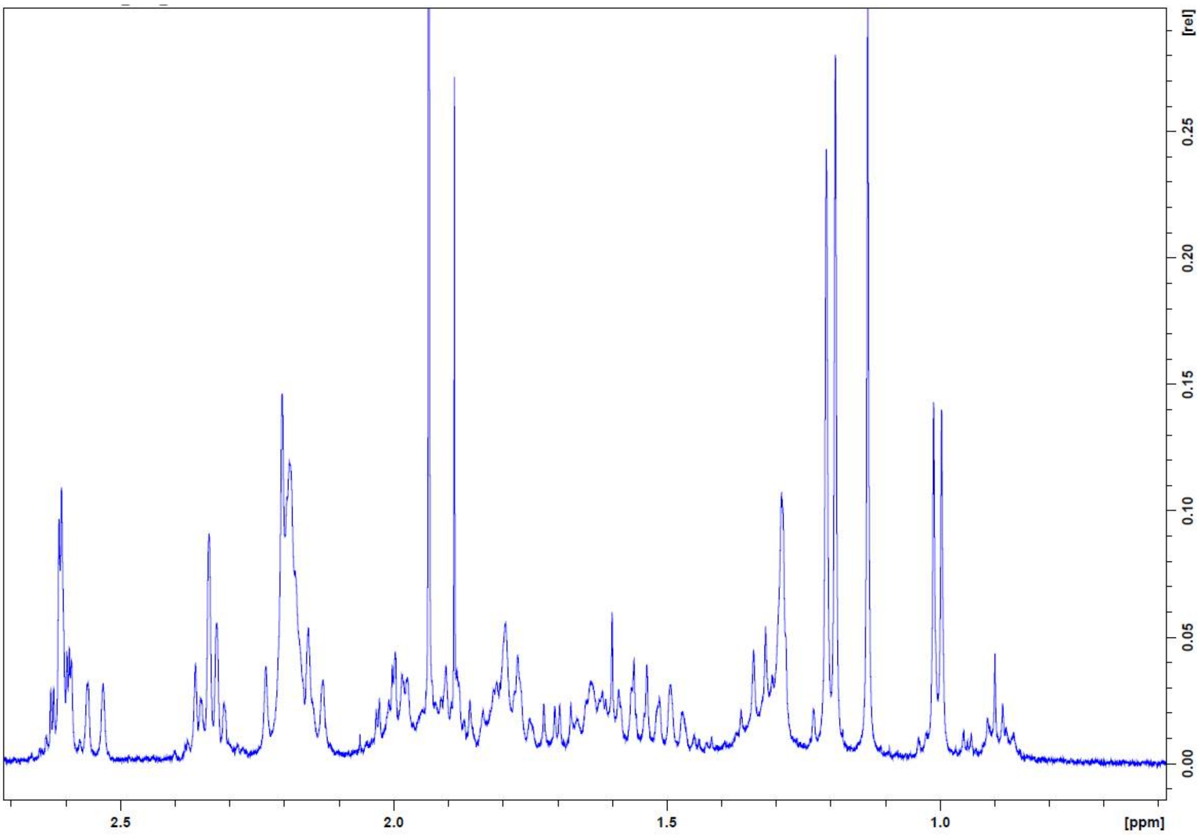

Figure S3. Expansion of the ^{1}H NMR spectrum (0.6–2.7 ppm) of gambierone in CD_3OD (≥99.8% atom D) at 500 MHz.

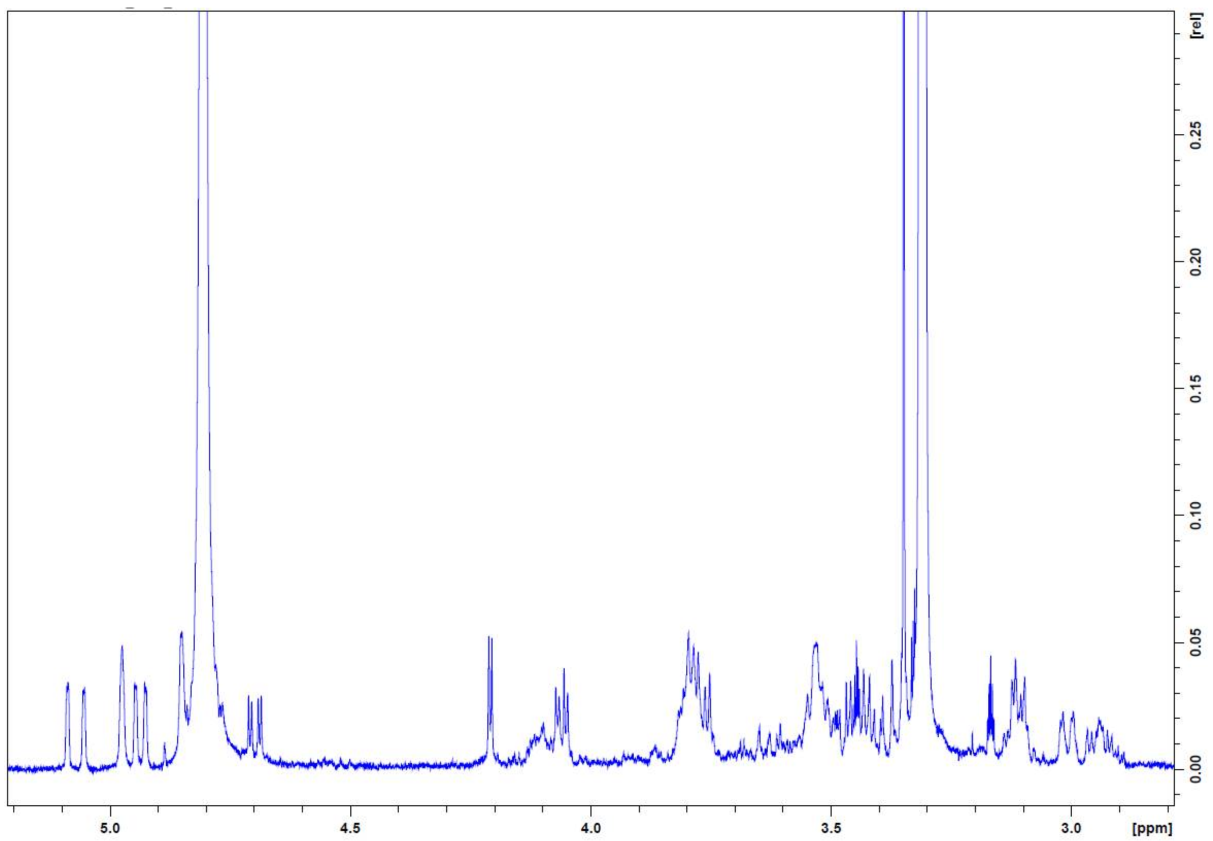

Figure S4. Expansion of the ^{1}H NMR spectrum (2.8–5.2 ppm) of gambierone in CD_3OD (≥99.8% atom D) at 500 MHz.

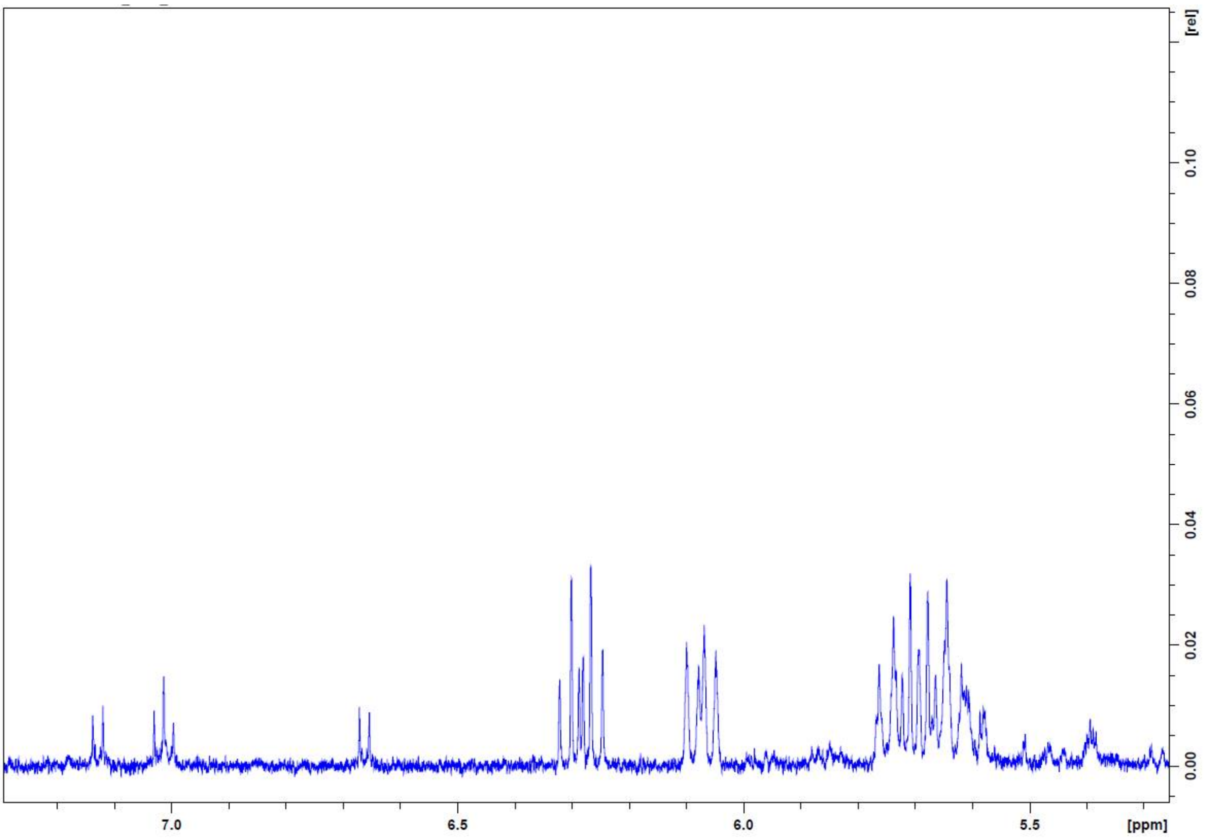

Figure S5. Expansion of the ^{1}H NMR spectrum (5.3–7.3 ppm) of gambierone in CD_3OD (≥99.8% atom D) at 500 MHz.

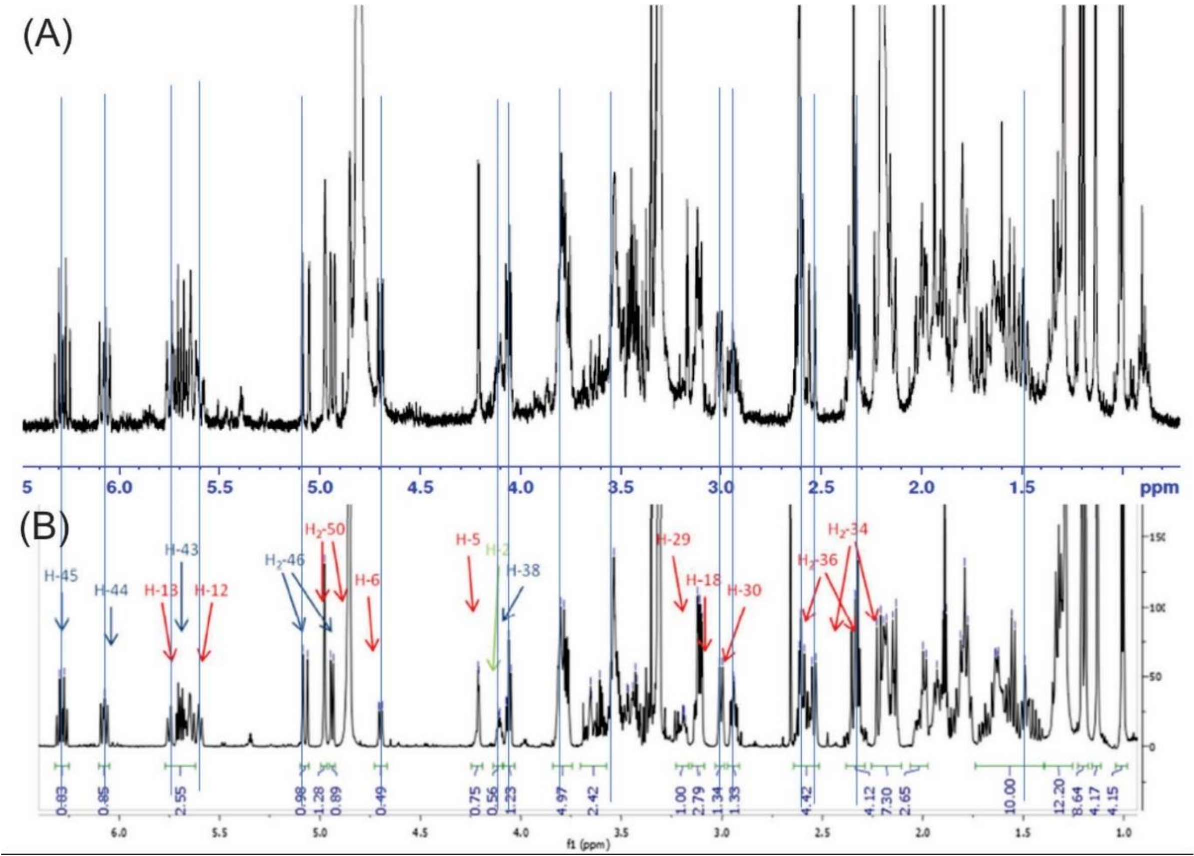

Figure S6. Comparison of the ^{1}H NMR spectrum of (**A**) gambierone purified from *Gambierdiscus cheloniae* CAWD232 acquired on a Bruker Advance III 500 MHz instrument and (**B**) the published spectrum from Rodriguez *et al*., acquired on a Varian Inova 750 MHz instrument [38].

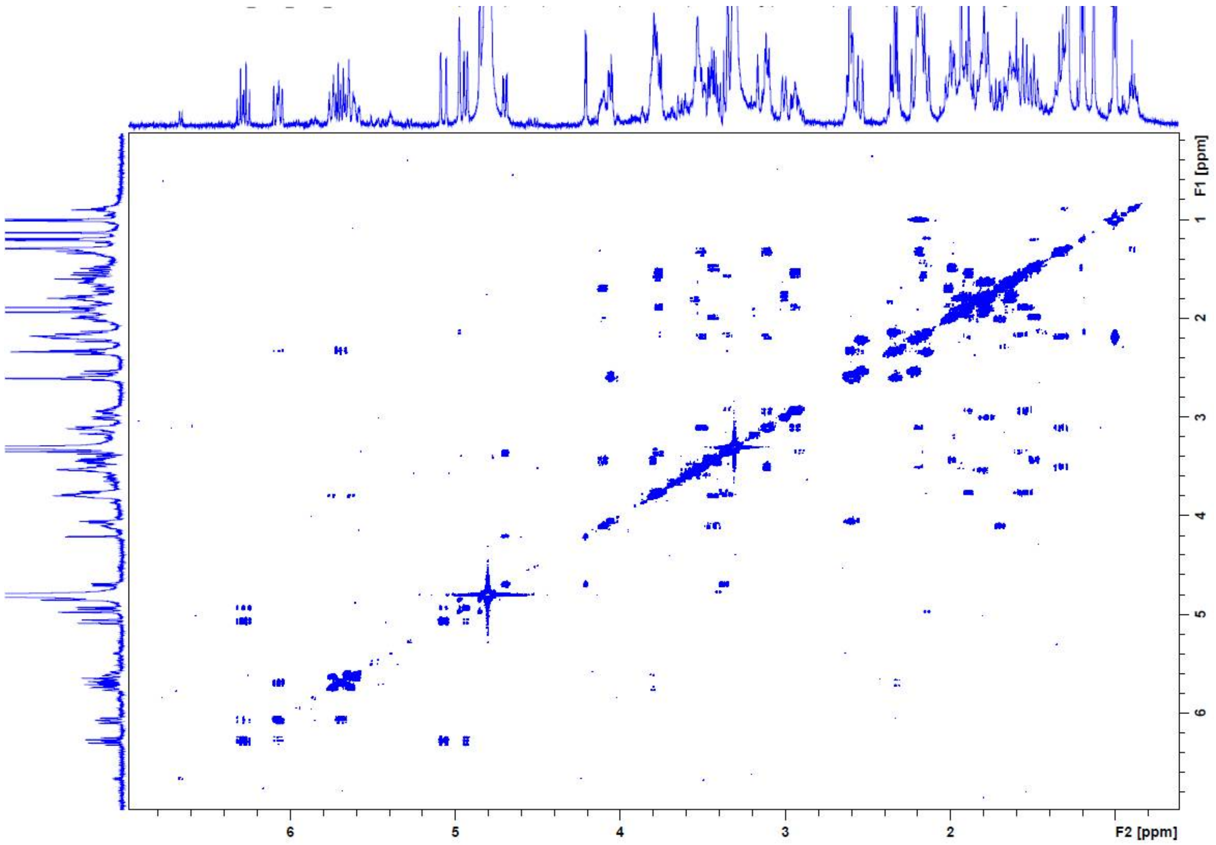

Figure S7. COSY NMR spectrum of gambierone purified from *Gambierdiscus cheloniae* CAWD232 in CD_3OD (≥99.8% atom D) at 500 MHz.

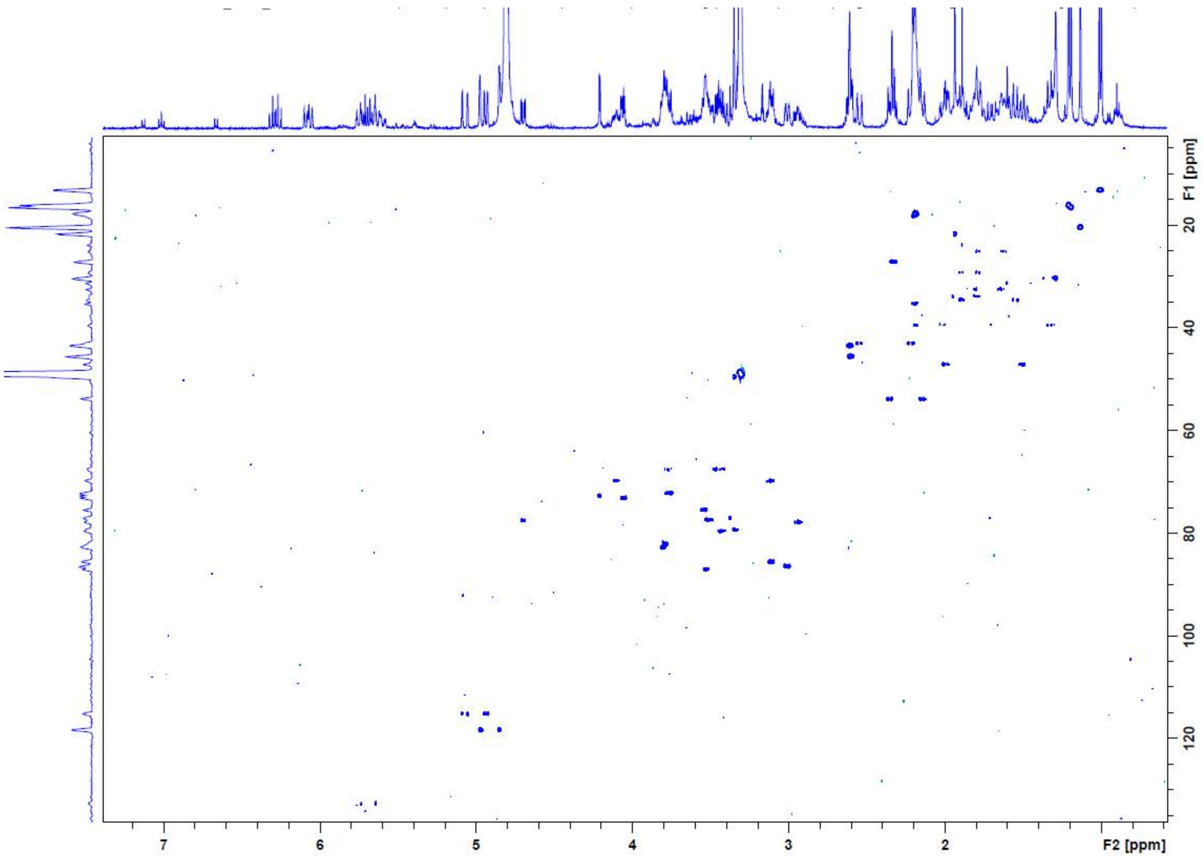

Figure S8. HSQC NMR spectrum of gambierone purified from *Gambierdiscus cheloniae* CAWD232 in CD_3OD (≥99.8% atom D) at 500 MHz.

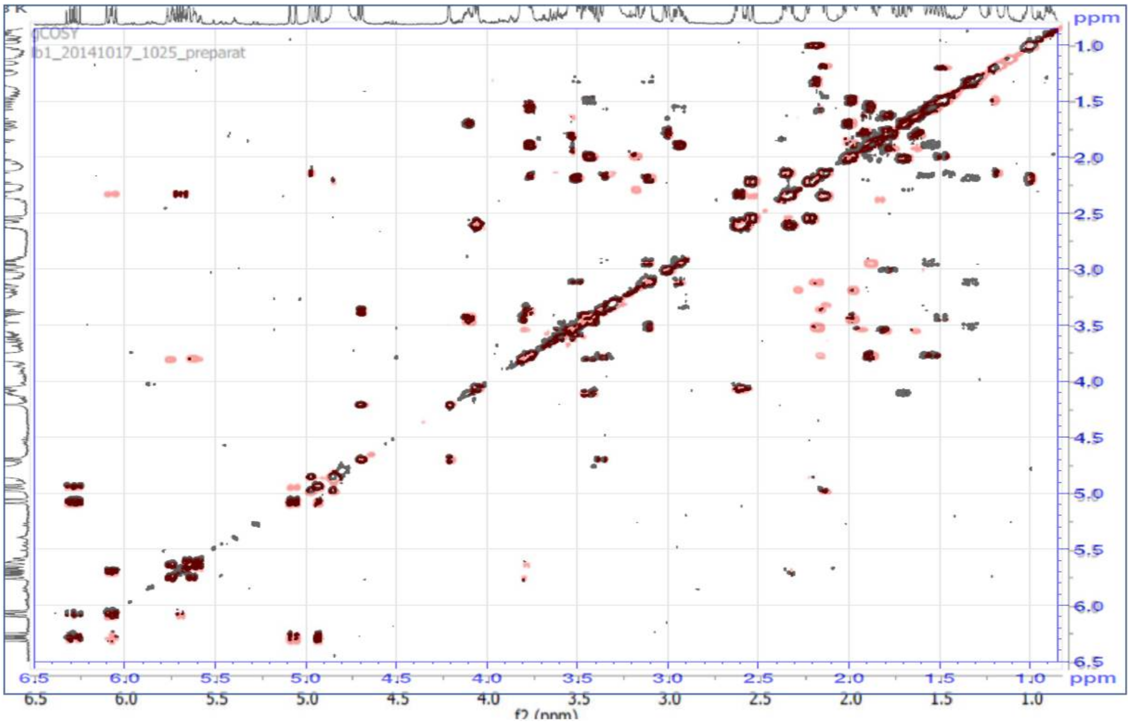

Figure S9. Comparison of the COSY NMR spectrum (1.0–6.5 ppm) of gambierone purified from *Gambierdiscus cheloniae* CAWD232 (black) and that published by Rodriguez *et al.*, 2015 (red) [38]. Long range (~2 Hz) couplings not displayed.

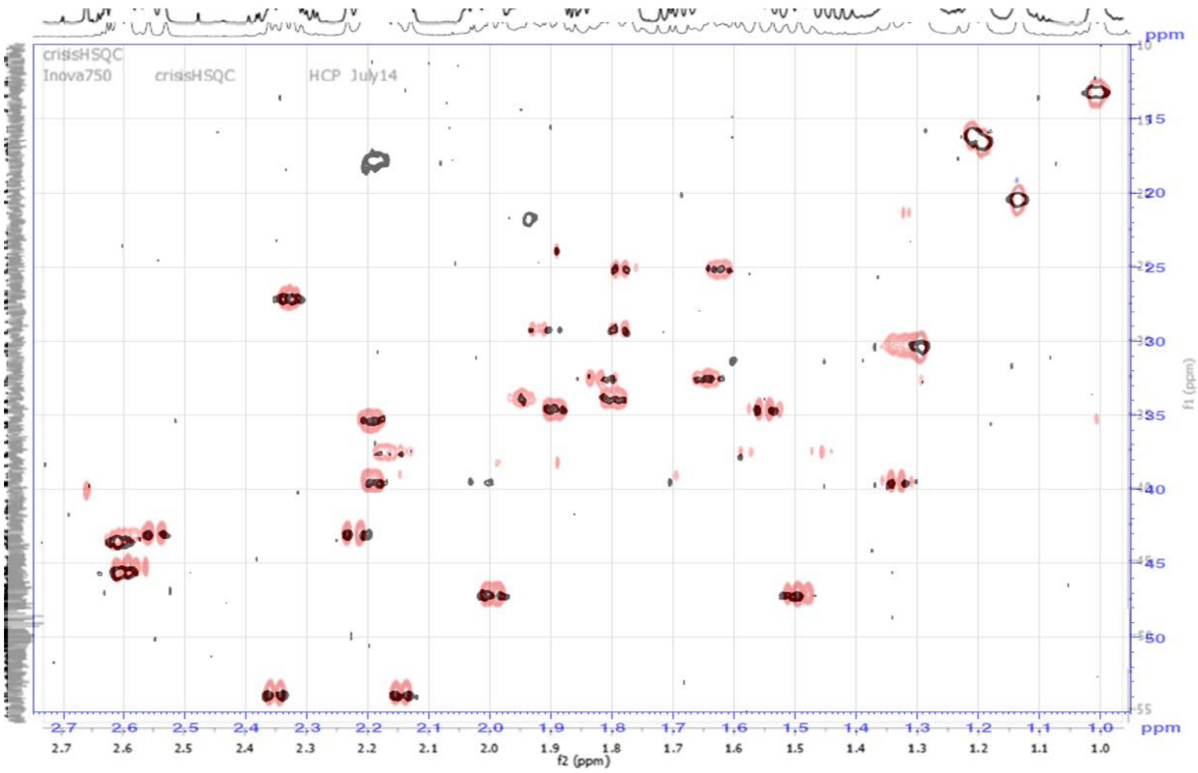

Figure S10. Expansion (^{1}H: 1.0–2.7 ppm; ^{13}C: 10–55 ppm) of the HSQC spectrum of gambierone purified from *Gambierdiscus cheloniae* CAWD232 (black), and the published spectrum from Rodriguez *et al*., 2015 (red) [38].

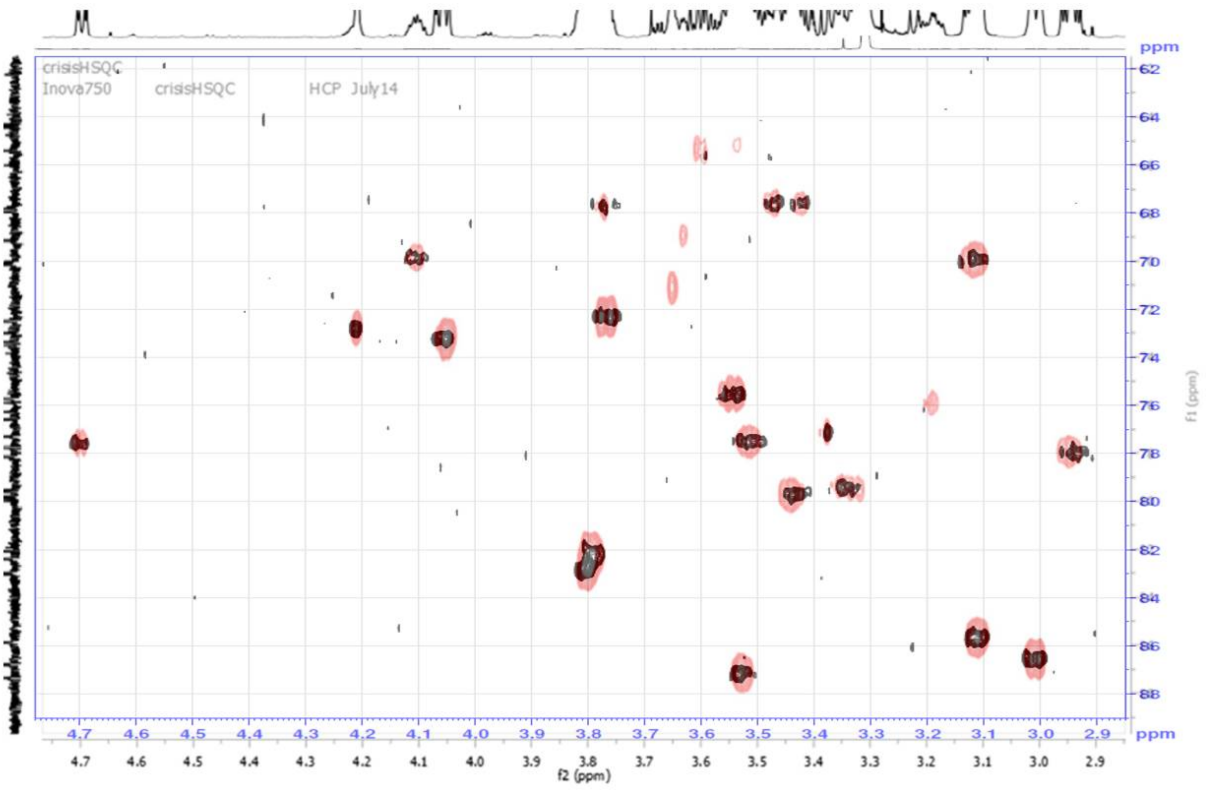

Figure S11. Expansion (^{1}H: 2.5–4.7 ppm; ^{13}C: 62–85 ppm) of the HSQC spectrum of gambierone purified from *Gambierdiscus cheloniae* CAWD232 (black), and the published spectrum from Rodriguez *et al*., 2015 (red) [38].

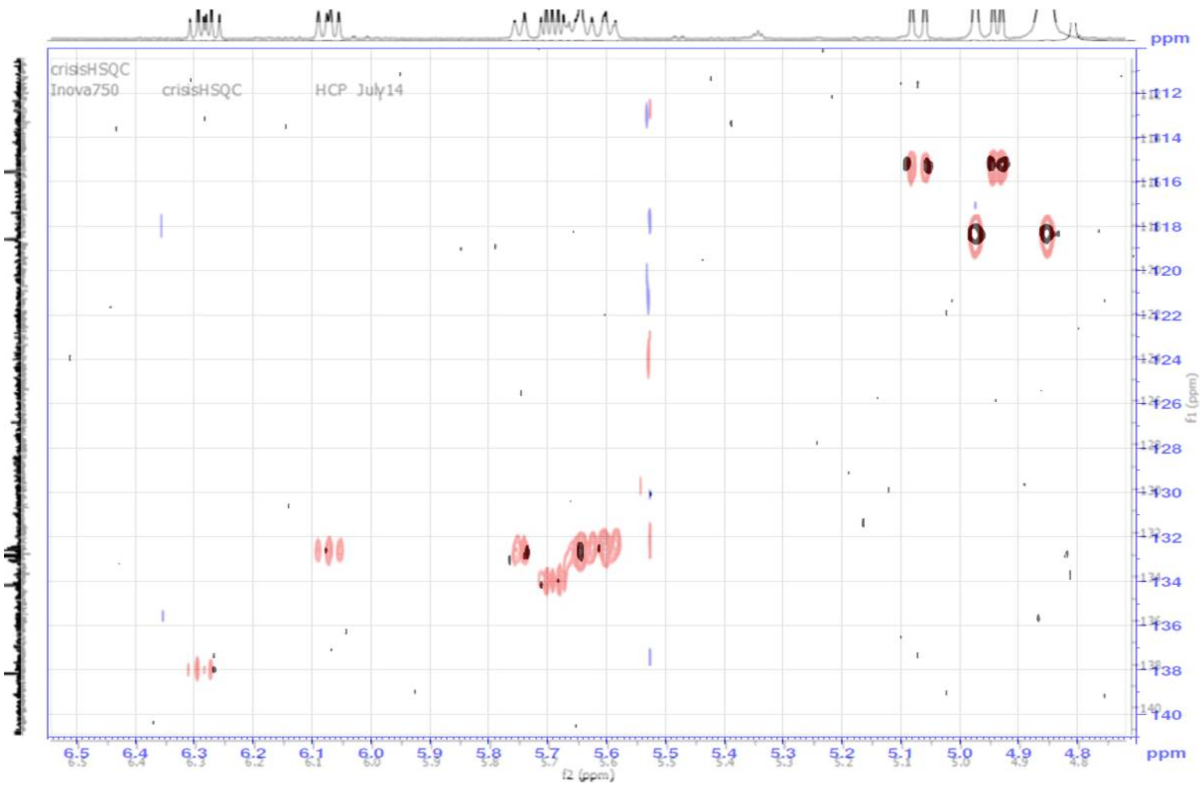

Figure S12. Expansion (^{1}H: 4.7–6.5 ppm; ^{13}C: 110–140 ppm) of the HSQC spectrum of gambierone purified from *Gambierdiscus cheloniae* CAWD232 (black), and the published spectrum from Rodriguez *et al*., 2015 (red) [38].

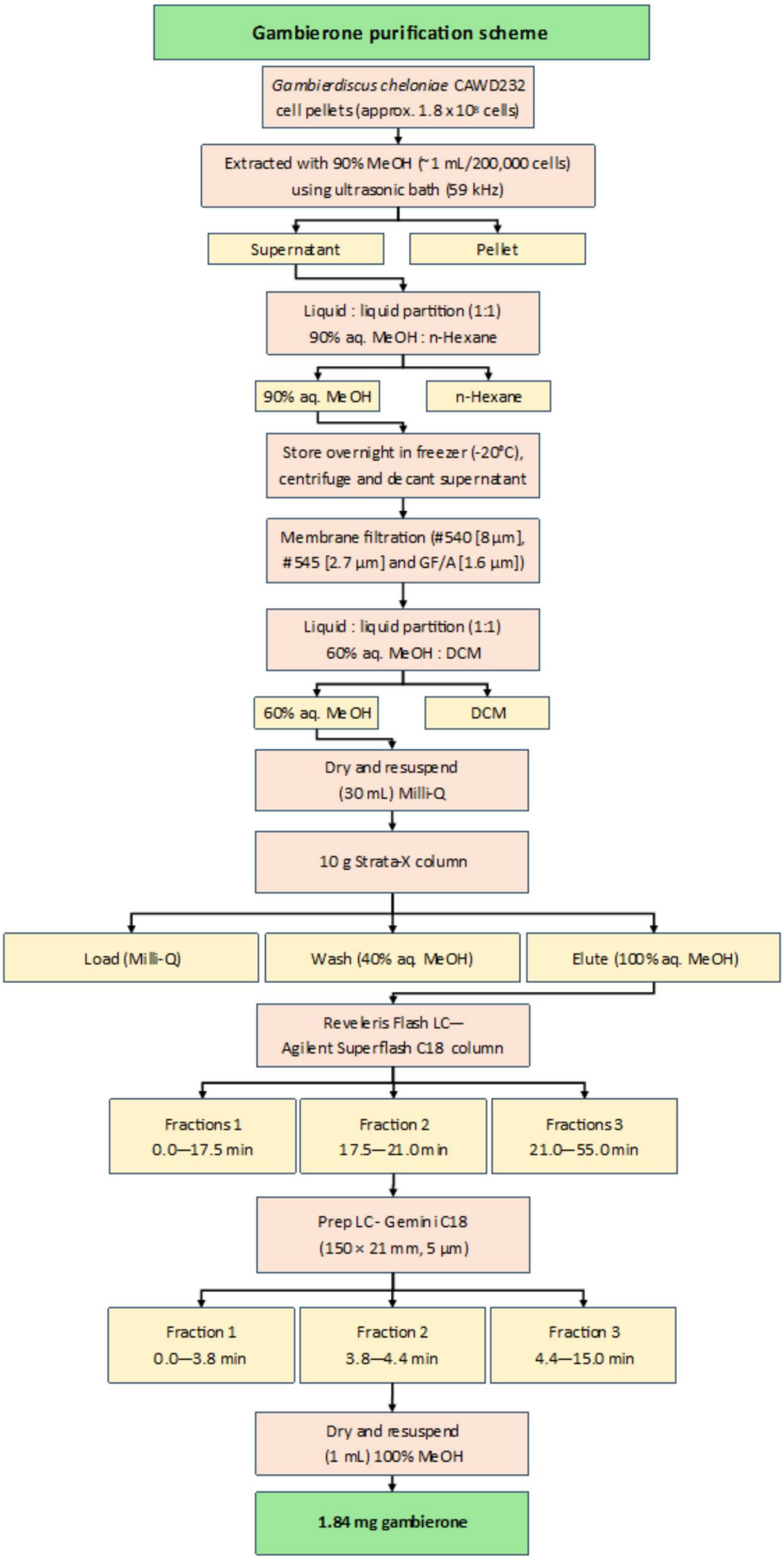

Figure S13. Purification scheme for the isolation of gambierone from *Gambierdiscus cheloniae* CAWD232.
